# Positive Crosstalk Between Hedgehog and NF-κB Pathways Is Dependent on KRAS Mutation in Pancreatic Ductal Adenocarcinoma

**DOI:** 10.3389/fonc.2021.652283

**Published:** 2021-05-11

**Authors:** Yuqiong Wang, Dan Wang, Yanmiao Dai, Xiangyu Kong, Xian Zhu, Yunxia Fan, Yaodong Wang, Hongyu Wu, Jing Jin, Wenzhu Yao, Jun Gao, Kaixuan Wang, Hongwei Xu

**Affiliations:** ^1^ Department of Gastroenterology, the Hospital of 92608 People's Liberation Army of China (PLA) Troops, Shanghai, China; ^2^ Institute of Oncology, Second Affiliated Hospital, Xi’an Medical University, Xi’an, China; ^3^ Department of Gastroenterology, Kunshan Affiliated Hospital of Nanjing University of Chinese Medicine, Suzhou, China; ^4^ Department of Gastroenterology, Changhai Hospital, Second Military Medical University, Shanghai, China; ^5^ Bureau of headmaster, Xi’an Medical University, Xi’an, China

**Keywords:** KRAS mutation, hedgehog pathway, NF-κB pathway, crosstalk mechanism, pancreatic ductal adenocarcinoma

## Abstract

It has been shown that aberrant activation of the Hedgehog (Hh) and nuclear factor-kappa B (NF-κB) signaling pathways plays an important role in the pancreatic carcinogenesis, and KRAS mutation is a hallmark of pancreatic ductal adenocarcinoma (PDAC). Until now, the role of KRAS mutation in the context of crosstalk between Hh and NF-κB signaling pathways in PDAC has not been investigated. This study was to determine whether the crosstalk between the Hh and NF-κB pathways is dependent on KRAS mutation in PDAC. The correlation between Gli1, Shh, NF-κB p65 expression and KRAS mutation in PDAC tissues was firstly examined by immunohistochemistry. Next, Western blotting, qPCR, and immunofluorescence were conducted to examine the biological effects of interleukin-1β (IL-1β) and tumor necrosis factor-alpha (TNF-α) as NF-κB signaling agonists, Shh as an Hh ligand alone or in combination with KRAS small interfering RNA (si-KRAS) in KRAS-mutant PDAC cells (MT-KRAS; SW1990 and Panc-1), wild-type KRAS PDAC cells (WT-KRAS; BxPC-3) and mutant KRAS knock-in BxPC-3 cells *in vitro* as well as tumor growth *in vivo*. KRAS mutation-dependent crosstalk between Hh and NF-κB in PDAC cells was further assessed by Ras activity and luciferase reporter assays. The aberrant Hh and NF-κB pathway activation was found in PDAC tissues with KRAS mutation. The same findings were confirmed in MT-KRAS PDAC cells and MT-KRAS knock-in BxPC-3 cells, whereas this activation was not observed in WT-KRAS PDAC cells. However, the activation was significantly down-regulated by KRAS silencing in MT-KRAS PDAC cells. Furthermore, MT-KRAS cancer cell proliferation and survival *in vitro* and tumor growth after inoculation with MT-KRAS cells *in vivo* were promoted by NF-κB and Hh signaling activation. The pivotal factor for co-activation of NF-κB and Hh signaling is MT-KRAS protein upregulation, showing that positive crosstalk between Hh and NF-κB pathways is dependent upon KRAS mutation in PDAC.

## Introduction

Pancreatic ductal adenocarcinoma (PDAC) is one of the most lethal malignancies, with an overall 5-year relative survival rate below 9% (1). Its development and progression are multistep processes involving numerous aberrant genes and signaling pathways (2). Based on global genomic analyses, 12 core signaling pathways and processes have been identified as having clear involvement in PDAC carcinogenesis, suggesting that deregulation of these core pathways are involved in the tumorigenesis of pancreatic cancer (3–5). Among these pathways, the Hedgehog (Hh), nuclear factor-kappa B (NF-κB), and KRAS signaling pathways have been the most studied and their aberrant activation has been shown to play an important role in the pancreatic carcinogenesis (6, 7).

Hh signaling was first discovered in the Drosophila fruit fly by Nusslein-Volhard and Wieschaus, and has since been linked to many aspects of tumor development and disease (8). Specifically, the Hh signaling pathway plays a critical role in pancreatic embryological development, but is inhibited in the mature pancreas. In addition, the Hh signaling pathway is aberrantly activated in the tumor microenvironment of pancreatic cancer to promote the growth of tumor stroma (9). Recent studies, including ours, have suggested that the activation of the Hh pathway has malignant characteristics of the canonical Hh pathway in the tumor stroma and non-canonical Hh-pathway in tumor cells (10–14). Sonic hedgehog (Shh) is the key molecule in the Hh signaling pathway, exerting its role by binding to the target cell member receptor. Shh can induce the activation of the Gli family of transcription factors including Gli1, by the classical pathway as well as the nonclassical pathway (15).

KRAS mutation is a hallmark of PDAC and is detected in 75–95% of these malignancies (16, 17). The role of aberrant KRAS signaling caused by mutation in pancreatic cancer has been clearly demonstrated in genetically engineered mouse models, with the oncogene KRAS G12D mutation conditionally activated in the embryonic pancreas (7). However, in pancreatic epithelial cells, the KRAS G12D activation can induce the development of pancreatic intraepithelial neoplasia (PanIN) precursor lesions, which eventually progress to invasive PDAC after a long period of latency (18–20). If the expression of oncogenic KRAS transgenes is silenced during pancreas development and only activated in adult animals, neither PanIN development nor tumor will develop (21). In addition, KRAS mutations arise presumably in the adult pancreas (2). In that case, KRAS mutation may facilitate or amplify the aberrant activation of other genes or pathways and their interactions, leading to PDAC progression (16, 22, 23).

Stromal hyperplasia is the distinct pathological feature of PDAC, which results in a pronounced pro-inflammatory microenvironment which is driven by the secretion of multiple tumor-derived pro-inflammatory cytokines for cancer cells (18, 24). Indeed, pivotal pro-inflammatory cytokines, including tumor necrosis factor-α (TNF-α) and interleukin-1β (IL-1β), may be secreted by tumor-associated macrophages to further promote tumor cell growth, migration, invasion, and the epithelial to mesenchymal transition, mainly *via* the NF-κB pathway (10, 24–26).

Our previous study indicated that pro-inflammatory cytokines, mainly TNF-α and IL-1β, played a key role in the activation of both canonical and non-canonical Hh pathways in an NF-κB activation-dependent manner in PDAC (10). Intrigued by and built upon these previous findings, we attempted to illuminate the specific mechanisms for the KRAS dependent mutual activation of NF-κB and Hh signaling in PDAC.

## Materials and Methods

### Patients and Samples

Forty patients diagnosed with PDAC and confirmed by histopathology were selected for this study. The study included 14 females and 26 males with a median age at surgery of 58.30 years (range, 50–65 years). Tissue samples were obtained from 2012–2014 at the time of surgery at Changhai Hospital (Shanghai, China). Informed consents were obtained from all patients, and the study was approved by Changhai Hospital Ethics Review Committee. Tissue samples were formalin-fixed and paraffin-embedded, sliced in 3 μm sections, and stained with hematoxylin and eosin (H&E) for routine pathological examination.

### Immunohistochemistry

For immunohistochemical (IHC) staining, tissue sections were deparaffinized in xylene, hydrated in a series of diluted ethanol, and subsequently immersed in 3% hydrogen peroxide for 10 min to inactivate endogenous peroxidases. After antigen retrieval, slides were incubated at 4°C overnight with primary antibodies, including rabbit anti-Gli1 polyclonal antibody (1:100, ab49314; Abcam), mouse anti-NF-κB p65 monoclonal antibody (1:50, SC-8008; Santa Cruz), or rabbit anti-Shh monoclonal antibody (1:200, ab53281; Abcam). The next day, slides were washed three times with phosphate-buffered saline (PBS) and incubated for 45 min at room temperature with the appropriate goat anti-rabbit secondary antibody (1:500, ab7090; Abcam) or goat anti-mouse secondary antibody (1:500, ab97040; Abcam). Then the slides were washed with PBS, immersed in liquid 3,3’-diaminobenzidine (DAB) for 5 min, and counterstained with hematoxylin. PBS instead of primary antibody was used for the negative controls. Nuclear expression of Gli1, NF-κB p65, and Shh expression were assessed independently by two pathologists. Staining intensity and distribution were quantified by semi-quantitative scoring. Staining intensity was defined as weak, moderate, or intense while staining distribution was defined as focal (≤10%), regional (11–50%), or diffuse (>50%).

### Cell Culture and Treatment

PDAC cell lines (SW1990, Panc-1, and BxPC-3) were purchased from the Type Culture Collection of the Chinese Academy of Sciences (Shanghai, China). Cells were cultured in Dulbecco’s Modified Eagle Medium (DMEM; Gibco, USA) supplemented with 10% fetal bovine serum (FBS; Gibco, USA) and 100 U/mL penicillin-100 ng/mL streptomycin solution (Hyclone, USA). Recombinant human sonic hedgehog (rh-Shh; 1845-SH-100), recombinant human IL-1β (rh-IL-1β; 201-LB-025), and recombinant human TNF-α (rh-TNF-α; 210-TA-100) were purchased from R&D Systems (USA). Human KRAS small interfering RNA (si-KRAS) was purchased from Dharmacon Company. In brief, 2×106 cells were seeded in 10 cm dishes (Corning, USA). When a 50% cell density was reached, cells were starved with 1% FBS-DMEM and then treated with 100 ng/mL Shh, 5 ng/mL IL-1β, 10 ng/mL TNF-α (in 1% FBS-DMEM), and si-KRAS alone or in combination for 48 h, while the medium was freshly replaced every 6 h.

### Detection of KRAS Mutations in Codons 12 and 13

For further testing of KRAS mutations, Genomic DNA was extracted from tissue samples using the DNeasy Blood & Tissue Kit (Qiagen, Germany). DNA quantification was done with the Qubit 2.0 Fluorimeter with dsDNA High Sensitivity Assay Kit (Thermo, Germany). Sequencing of KRAS codons 12/13 was performed by Shanghai Life Technologies Biotechnology Co., Ltd in China.

### RNA Extraction and Quantitative PCR

For analysis of mRNA expression, total RNA was extracted from PDAC cells using trizol reagent and purified through digestion with DNase I for 15 min. RNA was recovered using the RNeasy Kit (Qiagen, Germany). Reverse transcription of 1 µg purified RNA was conducted in a 20 µL reverse transcription system for 15 min at 37°C according to the manufacturer’s protocol. Gli1, NF-κB, Shh, KRAS, and GAPDH Taqman primers were purchased from Invitrogen (Shanghai Life Technologies Biotechnology Co., Ltd.). For quantitative PCR (qPCR), 20 μL mixture reactions including gene-specific taqman primers were run the Roche LightCycler^®^ 480 instrument (Roche, Switzerland). The amount of each target gene in a given sample was normalized to the level of GAPDH in that sample. All experiments were run in triplicate.

### Extraction of Nuclear and Cytoplasmic Proteins and Western Blotting

For protein analysis, adherent cells were collected with 0.25% trypsin (Gibco; USA) and centrifuged at 1,000 rpm for 5 min. Next, cells were washed twice with PBS and nuclear and cytoplasmic proteins were extracted with the NE-PER Nuclear and Cytoplasmic Extraction Reagents Kit (Thermo, USA). Protein concentration was then determined using the bicinchoninic acid (BCA) protein assay kit (Thermo, USA) according to the manufacturer’s instructions. Both nuclear and cytoplasmic proteins were separated on sodium dodecyl sulfate-polyacrylamide gel electrophoresis (SDS-PAGE), transferred to 0.45 μm polyvinylidene difluoride membranes (PVDF, Bio-Rad; USA), and blocked with 1% bovine serum albumin for 1 h. Then, membranes were incubated overnight at 4°C with rabbit anti-Shh monoclonal antibody (1:2000, ab53281; Abcam), rabbit anti-Gli1 polyclonal antibody (1:1000, ab49314; Abcam), mouse anti-NF-κB p65 monoclonal antibody (1:500, SC-8008; Santa Cruz), rabbit anti-KRAS polyclonal antibody (1:1000, ab180772; Abcam), rabbit anti-GAPDH polyclonal antibody (1:2000, ab9485; Abcam), rabbit anti-phosphorylated ERK1/2 (p-ERK1/2) (1:2000, #4376; CST) and anti-total ERK1/2 (t-ERK1/2) monoclonal antibodies (1:2000, #4695; CST), rabbit anti-phosphorylated AKT1 (p-AKT1) (1:1000, #9018; CST) and anti-total-AKT1 (t-AKT1) monoclonal antibodies (1:1000, #2938; CST) and rabbit anti-histone H3 polyclonal antibody (1:3000, ab1791; Abcam), followed by secondary antibody with a dilution of 1:2000 (goat anti-rabbit antibody, ab7090, Abcam; goat anti-mouse antibody, ab97040, Abcam). The amount of each target gene was normalized to GAPDH or histone H3 in each sample. The blots were visualized using an enhanced chemiluminescence reagent (Thermo, USA).

### Immunofluorescence Analysis

For immunofluorescence analysis, SW1990, Panc-1 and BxPC-3 cells were seeded in 24-well plates containing a sterile glass slide. Cells were treated with different stimuli: 100 ng/mL Shh, 5 ng/mL IL-1β, 10 ng/mL TNF-α (in 1% FBS-DMEM) and si-KRAS alone or in combination for 48 h, and then washed three times with ice-cold PBS, fixed in 4% paraformaldehyde for 20 min at room temperature, and finally treated with 0.1% triton for 30 min. After blocking in 1% bovine serum albumin for 1 h at room temperature, the slides were incubated at 4°C overnight with the indicated primary antibody, including rabbit anti-Gli1 (1:100, ab49314; Abcam), rabbit anti-NF-κB p65 subunit (1:50, SC-8008; Santa Cruz) and rabbit anti-Shh (1:200, ab53281; Abcam). Subsequently, fluorescein Dylight 488 or Dylight 594 conjugated donkey anti-rabbit secondary antibody (Jackson Laboratories, USA) at a dilution of 1: 200 was added into cells for 1 h at room temperature. Finally, slides were washed and examined under a fluorescence microscope (Olympus, Japan).

### Ras Activity Assay

Ras activity was measured using Ras Activation Assay Kit (Millipore, USA) according to the manufacturer’s protocol. Briefly, cells were homogenized in 1× lysis buffer on ice, then sonicated and centrifuged at 12,000 rpm at 4°C for 10 min. Protein concentration was determined by the BCA Protein Assay Kit. Then 1/10 of the lysate was set aside to allow quantification of total Ras and protein concentration. GAPDH was used as an internal control. Equal amounts of lysate were incubated for 1 h at 4°C with agarose beads coated with the Raf-Ras binding domain provided in the kit. Beads were washed three times with ice-cold lysis buffer, and then boiled for 5 min with loading buffer.

### Luciferase Assay

For the luciferase assay, cells in 6-well plates were co-transfected with 10 ng pRL-TK (Promega, USA) and 500 ng pGL3-8×Gli1 binding site (BS) luciferase reporter plasmids (wild-type, WT or mutant-type, MT) by FuGENE HD Transfection (Promega, USA). After 8 h, the medium was changed to 1% FBS-DMEM (control), 100 ng/mL Shh, 5 ng/mL IL-1β, or 10 ng/mL TNF-α in 1% FBS-DMEM for treatment. Luciferase assays were performed 48 h later according to the protocol of the Dual Luciferase Assay Kit (Promega, USA). The luciferase activities were normalized to the Renilla luciferase activity. MT pGL3-8×Gli1 BS luciferase report plasmids were used as control. pGL3-8×Gli1 BS luciferase reporter plasmids (WT or MT) were kindly gifted by Doctor Lei Li from the Shanghai Changhai Hospital.

### Cell Counting Kit-8 Assay

Cell viability was determined using the Cell Counting Kit-8 Assay Kit (Dojindo, Japan). Briefly, cells were seeded into 96-well plates at a density of 2×104 cells per well overnight and treated with 1% FBS-DMEM, 100 ng/mL Shh, 5 ng/mL IL-1β, 10 ng/mL TNF-α or si-KRAS (0.15 ng) transfected alone or in combination. At 0, 24, 36, and 48 h post-stimulation, cells were treated with 2-(2-methoxy-4-nitrophenyl)-3-(4-nitrophenyl)-5-(2,4-disulfophenyl)-2H-tetrazolium mono-sodium salt (WST-8) in the dark according to the protocol. Absorbance values were measured at 450 nm by an enzyme-linked immunosorbent assay. All experiments were performed in triplicate.

### Apoptosis Assay

Apoptosis was detected and quantified using the Annexin V/FITC staining kit (eBioscience, USA). At 48 h after stimulation, cells were collected and washed twice with PBS. Then cells were centrifuged, resuspended in 100 μL binding buffer, and stained with Annexin V (5 μL) for 15 min at room temperature in the dark. Next, samples were stained with propidium iodide (10 μL) for 5 min in the dark before flow cytometry (Miltenyi Biotec, Germany). The results were analyzed by FlowJo software (Tree Star Inc., USA).

### MT-KRAS Plasmid Transfection

MT-KRAS plasmid (KRAS (G12D) mutation) was purchased from Addgene (Watertown, USA). Cells in 6-well plates were transfected with MT-KRAS plasmid using FuGENE HD Transfection Reagent (Promega, USA). Specifically, cells in each well were transfected with 2 μg plasmid, and vehicle was used as control. After 8 h, cells were treated with 1% FBS-DMEM, 100 ng/mL Shh, 5 ng/mL IL-1β, or 10 ng/mL TNF-α in 1% FBS-DMEM. After 48 h, cells were collected for further analysis.

### Mouse Xenograft Models

All animal procedures were approved by the Institutional Animal Care and Use Committee of Shanghai Changhai Hospital and conducted in accordance with NIH guidelines for the care and use of laboratory animals. Four-week-old male athymic nude mice were purchased from the Shanghai Laboratory Animal Center in China and raised under specific pathogen-free conditions. SW1990, Panc-1, and BxPc-3 cells (4×106) were respectively resuspended in 100 μL PBS, and then injected subcutaneously into the right flank of mice. After mice developed tumors (for SW1990 and BxPC-3 after 1 week, and for Panc-1 after around 4 weeks), they were randomly assigned into control group (n = 5), Shh group (n = 5), IL-1β group (n = 5), or TNF-α group (n = 5). The administered treatments were as follows: the control group received 200 μL PBS intraperitoneally (ip) daily (QOD); the Shh group received 300 ng Shh (200 μL ip QOD); the IL-1β group received 10 ng IL-1β (200 μL ip QOD); and the TNF-α group received 500 ng TNF-α (200 μL ip QOD) (27–29). After 2 weeks, mice were sacrificed and tumor volumes (V) were calculated (V = [L × W2] × 0.5, [L - length, W - width]) (30). Next, tumor tissues were fixed in formalin for IHC staining, and staining intensity was scored as follows: 0, no staining; 1, mild staining, weak yellow; 2, moderate staining, dark yellow; 3, strong staining, brown; 4, very strong staining, dark brown. The percentage of stained cells was also determined. Finally, the immunoscore was obtained by calculating the product of the proportion of positive cells at each intensity and the corresponding intensity value (31, 32).

### Statistical Analysis

All calculations were performed using the Statistical Package for the Social Sciences (version 18.0; USA). The Student’s t-test was used to compare the groups and determine the statistical significance. Pearson’s correlation was used to calculate the correlation coefficient. All data are expressed as the mean ± standard deviation. P < 0.05 was considered statistically significant.

## Results

### Positive Correlation of Hh and NF-κB Pathway Activation on the Presence of KRAS Mutations in PDAC Tissues

**Figure 1:**
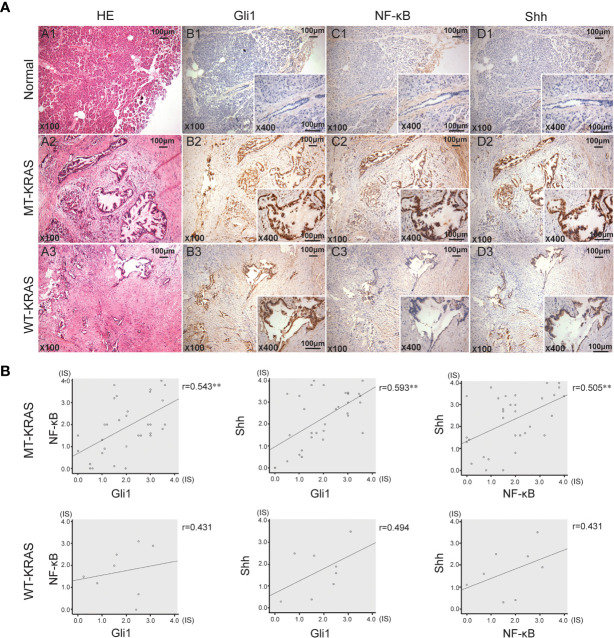
Gli1, NF-κB p65 and Shh protein expression in PDAC tissues with or without KRAS mutation. **(A)** Typical serial PDAC tissue section. HE staining and IHC staining for Gli1, NF-κB p65 and Shh proteins in the normal tissue (A1, B1, C1 and D1), MT-KRAS PDAC tissues (A2, B2, C2 and D2) and WT-KRAS PDAC tissues (A3, B3, C3 and D3). **(B)** Correlation of Gli1, NF-κB p65 and Shh protein expressions in the MT-KRAS and WT-KRAS PDAC tissues. **p < 0.01, n=40.

In 40 PDAC tissue samples, KRAS codons 12 and 13 were sequenced and the IHC expression of Shh, Gli1, and NF-κB p65 was examined. KRAS mutation (MT-KRAS) in 32 PDAC tissue samples was detected and no mutation (WT-KRAS) in 8 PDAC tissue samples was detected by sequencing technology. Meanwhile, a significant positive correlation in Gli1, Shh, and NF-κB p65 expression in MT-KRAS tissues was observed, whereas there was no correlation in WT-KRAS tissues by IHC staining (**Figure 1**). These results suggest that there is a KRAS mutation-dependent positive correlation in aberrant activation of Hh and NF-κB pathways in PDAC.

### Crosstalk Between Hh and NF-κB Pathways Relies on KRAS Mutation as a Type of Switch in PDAC Cells

**Figure 2:**
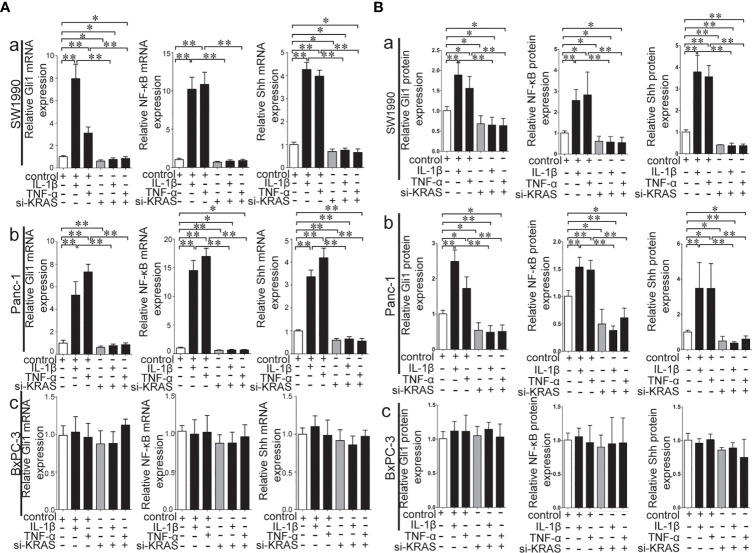
Stimulatory effects on mRNA and protein expression of Gli1, NF-κB and Shh on PDAC cell lines treated with IL-1β and TNF-α alone or in combination with si-KRAS. **(A)** represented mRNA expression in three PDAC cell lines; **(B)** represented gray analysis of relative protein expression; [a. SW1990, b Panc-1, c BxPC-3]. All data were obtained from three independent experiments. *p < 0.05; **p < 0.01.

**Figure 3:**
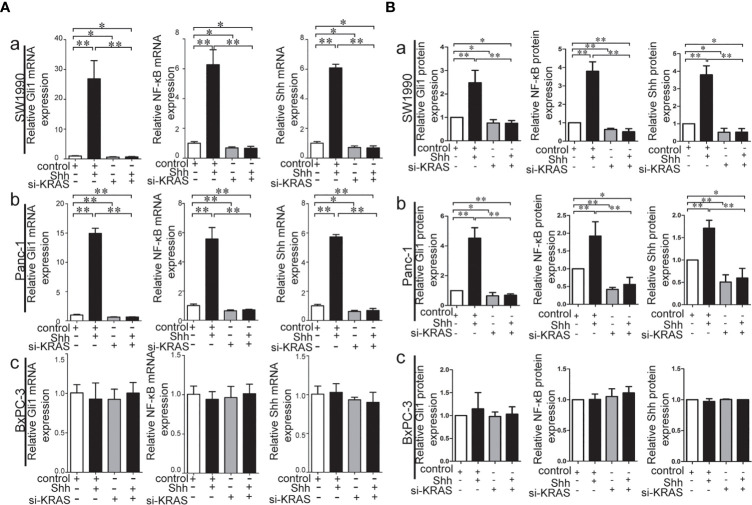
Stimulatory effects on mRNA and protein expression of Gli1, NF-κB and Shh on PDAC cell lines treated with Shh as Hh activating ligand treatment alone or in combination with si-KRAS transfection. **(A)** mRNA expression of Gli1, NF-κB and Shh in three PDAC cell lines; **(B)** Gray analysis of relative protein expression [a. SW1990, b. Panc-1, c. BxPC-3]. All data were obtained in three independent experiments. *p < 0.05; **p < 0.01.

**Figure 4:**
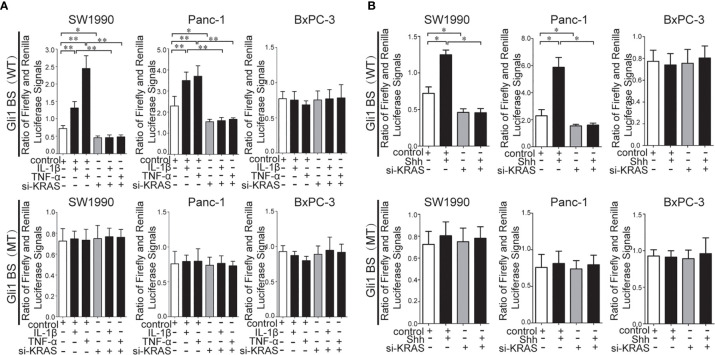
Results of the pGL3-8×Gli1 BS luciferase reporter assay in the MT-KRAS cells (SW1990 and Panc-1) and WT-KRAS cells (BxPC-3) after treatment with: IL-1β, TNF-α alone or in combination with si-KRAS transfection **(A)**. Shh alone or in combination with si-KRAS transfection **(B)**. All data were obtained in three independent experiments. *p < 0.05; **p < 0.01.

To further determine whether crosstalk between Hh and NF-κB pathways is indeed dependent on the presence or absence of KRAS mutation, three different PDAC cell lines (SW1990, Panc-1, and BxPC-3) were chosen based on their specific traits. SW1990 and Panc-1 cell lines have the G12D KRAS mutation whereas the BxPC-3 cell line is negative for KRAS mutation (**Figure S1**). In addition, Shh, Gli1, and NF-κB p65 mRNA and protein expression were detected in all cell lines (**Figure S2**). In this study, cells were treated with IL-1β, TNF-α and Shh and different responses were observed based on the presence or absence of KRAS mutation. Treatment with IL-1β and TNF-α, NF-κB signaling agonists, significantly upregulated NF-κB p65, Shh, and Gli1 mRNA [**Figures 2A(a, b)**], nuclear Gli1 and NF-κB protein, and Shh protein [**Figures 2B (a, b)**] expression in SW1990 and Panc-1 cells. After we demonstrated that si-KRAS transfections are effective in downregulating KRAS expression in the chosen cell lines (**Figure S4**), the combined treatment of si-KRAS with either IL-1β and TNF-α significantly downregulated the stimulatory effects caused by either stimulus alone [**Figures 2A(a, b)** and **2B (a, b)**]. However, no significant changes in the mRNA and protein expression of Gli1, NF-κB, and Shh were observed in BxPC-3 cells treated with IL-1β, TNF-α, or the combination of si-KRAS with either of these two cytokines [**Figures 2A(c)** and **2B(c)**]. The above original Western blots images were shown in **Figure S3**. Next, Shh as an Hh signaling ligand not only significantly upregulated the mRNA and protein expression of Shh and nuclear Gli1 but also NF-κB p65 mRNA and nuclear protein expression in SW1990 and Panc-1 cells, whereas the combined treatment with si-KRAS significantly abrogated the observed stimulatory effects [**Figures 3A(a, b), 3B(a, b)**]. However, no significant changes in Gli1, NF-κB, and Shh mRNA and protein expression were observed in BxPC-3 cells treated with Shh alone or in combination with si-KRAS [**Figures 3A(c), 3B(c)**]. The above original Western blots images were shown in **Figure S5** Similar effects on NF-κB p65 expression were observed with combined treatment of Shh and si-KRAS as detected by immunofluorescence staining in MT-KRAS cell lines SW1990 and Panc-1 and WT-KRAS cell line BxPC-3 (**Figure S6**). In addition, the pGL3-8×Gli1 BS luciferase reporter assay showed that the observed Gli1 gene promoter enhancement after this combined treatment was due to upregulated Gli1 gene expression in MT-KRAS cell lines SW1990 and Panc-1 whereas no significant changes were observed in BxPC-3 (**Figure 4**). To determine whether introduction of KRAS mutation in WT-KRAS PDAC cells could result in positive crosstalk between Hh and NF-κB signaling pathways in a similar manner as seen in MT-KRAS cells, an expression plasmid with KRAS (G12D) mutation (MT-KRAS) was constructed and its potency was validated in BxPC-3 cells (**Figure S7**). Subsequently, transfected cells were treated with IL-1β, TNF-α, or Shh. The results showed that IL-1β and TNF-α could upregulate Gli1 and Shh mRNA expression, and Shh stimulation enhanced NF-κB p65 mRNA expression in BxPC-3 transfected cells (**Figure S8**). But no significant protein expression changes were observed (Data not shown). These results suggest that the KRAS mutation knock-in can confer the WT-KRAS cell line BxPC-3 with similar traits as those of MT-KRAS cell lines SW1990 and Panc-1. These data indicate the positive crosstalk between Hh and NF-κB signaling pathways in PDAC cells, which is dependent upon the gain-of-function KRAS mutation.

### KRAS Protein Activity Is Essential for the Positive Interaction Between Hh and NF-κB Signaling Pathways

**Figure 5:**
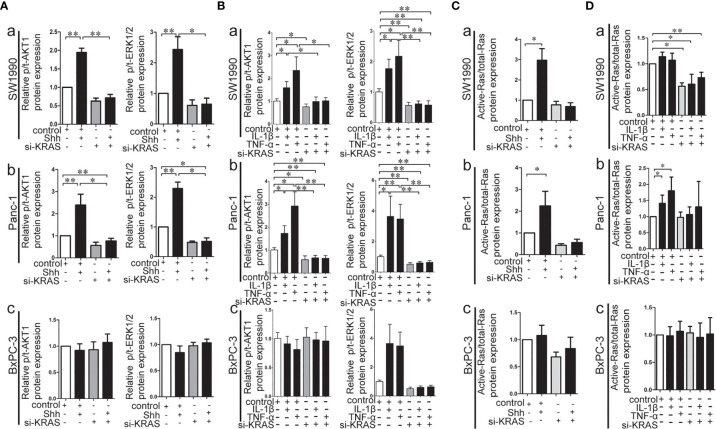
The effect of IL-1β, TNF-α, Shh treatment in combination with si-KRAS on KRAS down-stream molecules (p-/t-ERK1/2 and p-/t-AKT1) and Ras enzymatic activity in the MT-KRAS cell lines (SW1990 and Panc-1) and WT-KRAS cell line (BxPC-3). The protein expression of p-/t-ERK1/2 and p-/t-AKT1 at Shh treatment in combination with si-KRAS treatment **(A)**; The effect of combined IL-1β or TNF-α with si-KRAS transfection on p-/t-ERK1/2 and p-/t-AKT1 **(B)**; Ras enzymatic activity at Shh treatment in combination with si-KRAS treatment **(C)**; The effect of combined IL-1β or TNF-α with si-KRAS transfection on Ras enzymatic activity **(D)** [a. SW1990, b. Panc-1, c. BxPC-3]. All data were obtained in three independent experiments. *p < 0.05; **p < 0.01.

KRAS protein acts like a binary molecular switch, and can regulate cell activities only when bound to guanosine triphosphate (GTP). Mutations in KRAS protein render it insensitive to the function of GTP and lead to its constitutive activation (33). Therefore, the alteration of KRAS downstream signaling, such as p-/t-ERK1/2 and p/t-AKT1, and the Ras enzymatic activity were detected in MT-KRAS cells (SW1990 and Panc-1) and WT-KRAS cells (BxPC-3) treated with IL-1β, TNF-α, or Shh alone or in combination with si-KRAS. The expression of p-/t-ERK1/2 and p-/t-AKT1 was increased in SW1990 and Panc-1 cells after either IL-1β, TNF-α, or Shh stimulation, whereas the stimulating effects were significantly decreased by si-KRAS transfection in the combination treatments [(**Figures 5A (a, b), 5B (a, b)**]. However, the effects of IL-1β, TNF-α, and Shh stimulation on KRAS downstream effector molecules were not clearly observed in BxPC-3 cells treated either with IL-1β, TNF-α, Shh alone or in combination with si-KRAS [(**Figures 5A (c), 5B (c)**]. In addition, the stimulatory effects of IL-1β, TNF-α, Shh, and si-KRAS as combination treatments on Ras enzymatic activity were similar to alterations in KRAS downstream effector molecules under the same stimulus (**Figures 5C, D**). Original Western blots images were shown in **Figure S9**. Taken together, these data indicate that KRAS protein activity caused by KRAS mutation is an essential factor for the positive interaction between Hh and NF-κB signaling pathways in PDAC cells.

### KRAS Mutation Supports the Malignant Features of PDAC Cells Promoted by IL-1β, TNF-α, or Shh Treatment

**Figure 6:**
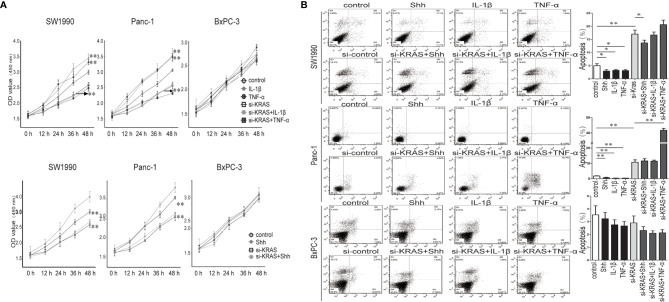
The proliferation and apoptosis effect on PDAC cell lines and PDAC tumor growth in a nude mouse xenograft tumor model treated IL-1β, TNF-α or Shh, in combination with si-KRAS transfection. **(A)** The effect on cell proliferation; **(B)** The effect on the apoptosis. *p < 0.05; **p < 0.01.

Next, we examined the effects of IL-1β, TNF-α, and Shh treatment alone or in combination with si-KRAS on the cellular processes of the three PDAC cell lines. As shown in **Figure 6**, in SW1990 and Panc-1 cells, malignant features such as cell proliferation and survival as indicated by apoptosis were significantly promoted by IL-1β, TNF-α, and Shh treatment compared with the controls. By contrast, malignant features were significantly less induced when cells were treated with those agents in combination with si-KRAS. Furthermore, although si-KRAS transfection in combination with those agents inhibited the features of malignant cells, treatment of SW1990 cells with Shh and Panc-1 cells with TNF-α still had a little effect on promoting malignant features such as proliferation and survival. However, all malignant features were unaltered in BxPC-3 cells treated with either IL-1β, TNF-α, Shh alone or in combination with si-KRAS, except for apoptosis in TNF-α treated Panc-1 cells, which may be caused by TNF-α specialty. These results suggest that the malignant features of PDAC cells are correlated with KRAS mutation, which acts through positive interaction between Hh and NF-κB signaling pathways.

### KRAS Mutation Induces PDAC Tumor Growth Promoted by IL-1β, TNF-α, and Shh Stimulation in a Xenograft Tumor Model

**Figure 7:**
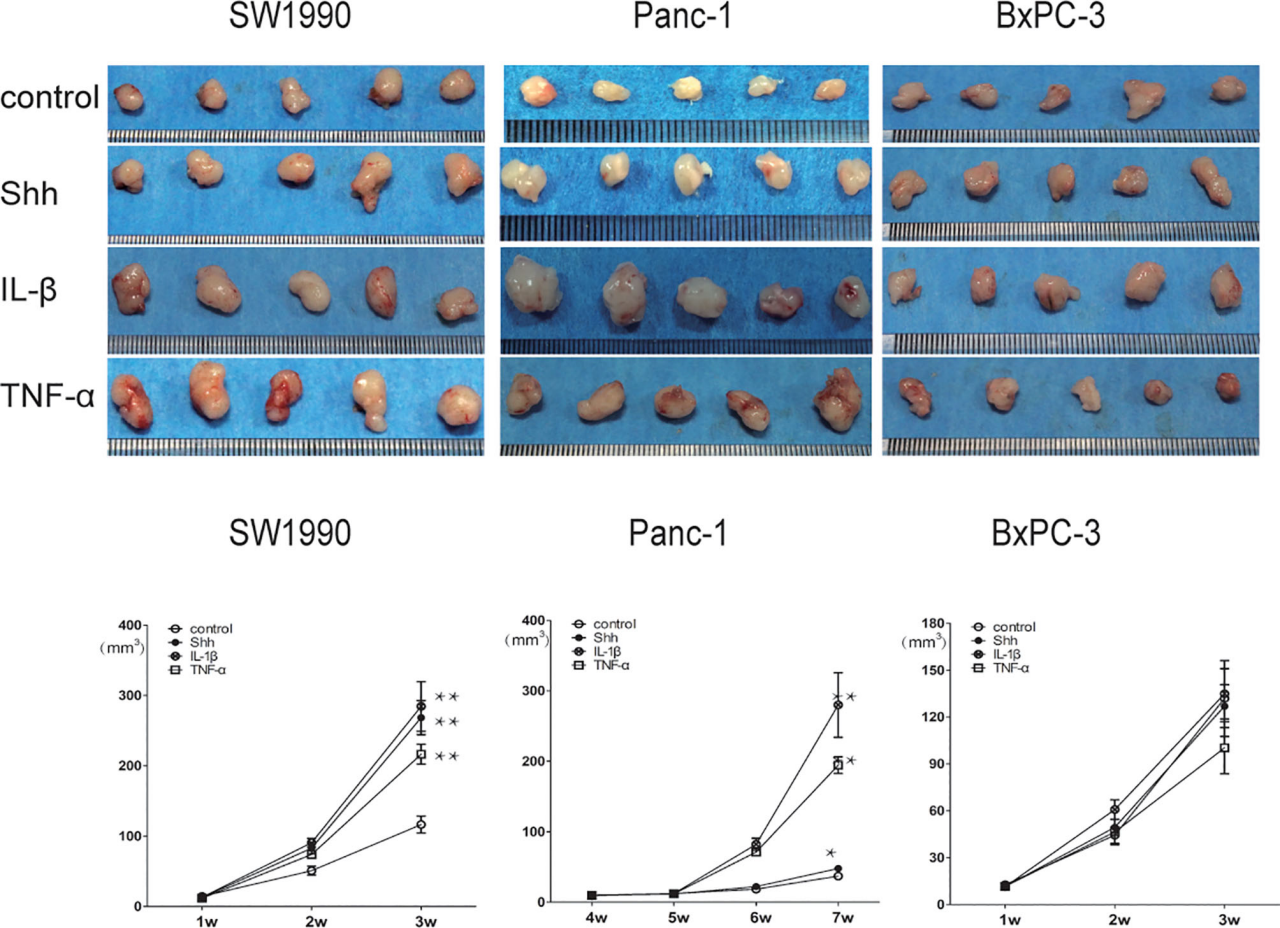
Tumor growth was significantly promoted by IL-1β, TNF-α, and Shh in mice inoculate d with MT-KRAS cells compared with those inoculated with WT-KRAS cells; *p < 0.05; **p < 0.01.

The nude mouse xenograft tumor model was used to further validate the KRAS mutation-dependent PDAC tumor growth promoted by IL-1β, TNF-α, and Shh stimulation. After mice were inoculated with specific PDAC cells and developed tumors, they were treated with PBS Shh, IL-1β, or TNF-α, and the tumor size was measured every week. After IL-1β, TNF-α, and Shh treatment, tumor growth from the MT-KRAS cell lines SW1990 and Panc-1 was significantly induced compared to the control animals, whereas tumor growth from BxPC-3 cells was not stimulated (**Figure 7**). Similarly, nuclear protein expression of Gli1 and NF-κB p65 was significantly enhanced in tumor tissues from SW1990 cells treated with IL-1β, TNF-α and Shh, whereas no significant expression was observed in the tissues from BxPC-3 cells (**Figure S10**). These results confirm that the dependence of PDAC tumor growth on KRAS mutation is promoted by positive crosstalk between Hh and NF-κB signaling pathways.

## Discussion

KRAS gene mutations are quite common in PDAC and appear early in pancreatic carcinogenesis (16). In our study, KRAS mutation was present in 80% (32 of 40) of PDACs. However, KRAS mutation alone in the mature pancreas is not enough to induce PDAC formation (21). In addition, a recent study showed that Hh signaling is required for KRAS-induced pancreatic tumorigenesis (34). Moreover, NF-κB expression is positively associated with Shh expression in PDAC, and NF-κB activation induces the upregulation of Hh signaling (6). In this study we stratified tumor samples according to the KRAS mutation status, and found that Hh signaling and NF-κB were positively correlated only in tumors with KRAS mutation. Thus, our findings support the importance of KRAS mutation in PDAC development, and demonstrate it in another yet not presented context of signaling crosstalk between the Hh and NF-κB pathways.

Based on the results in PDAC tissues, we assumed that KRAS mutation may provide a facilitating environmental milieu for the aberrant activation and interaction of signaling pathways in the stromal cells as well. Desmoplasia or dense fibrotic stroma is atypical feature of PDAC tissues, and stromal activation is usually found around precancerous lesions (35). Indeed, stromal tissue is not a mere bystander in tumor progression since it has been established that tumor–stroma interactions affect tumorigenesis, angiogenesis, therapy resistance, and possibly the metastatic spread of tumor cells (35). Cells that constitute the tumor stroma provide many factors to initiate and/or support tumorigenesis, including the pro-inflammatory cytokines IL-1β and TNF-α.

Whether Hh pathway activation can up-regulate NF-κB expression has rarely been reported, and the influence of KRAS mutation in this context has not been explored. To verify our hypothesis, we first used rh-IL-1β and rh-TNF-α to stimulate PDAC cells, and tested Hh pathway expression in cell lines with different KRAS mutation status. Our data showed that only in cells with KRAS mutation, rh-IL-1β and rh-TNF-α could initiate Hh pathway activation. These findings suggest that KRAS mutation is a necessary condition for NF-κB/Hh pathway interaction, in keeping with our results obtained from patients’ tumor samples.

Embryonic patterning and organ morphogenesis can be regulated by Hh signaling, which is also involved in the regeneration and repair of tissues in normal physiological conditions (11). Aberrant Hh pathway activation is a hallmark of PDAC. Hh pathway activation is mostly induced by Hh ligands that can generate tumor-stromal signaling crosstalk in an autocrine or paracrine manner (36). To further explore the relationship between the Hh pathway and NF-κB, we used rh-Shh to stimulate all three PDAC cell lines. Not surprisingly, NF-κB activation induced by Hh overexpression was only achieved in cell lines with KRAS mutation.

To confirm the necessity of KRAS mutation for Hh/NF-κB interplay in PDAC, we performed knocked down KRAS gene expression in all PDAC cell lines and constructed a knock-in of MT-KRAS plasmid in WT-KRAS cells. Our results showed that KRAS downregulation almost blocked the Hh/NF-κB interplay in MT-KRAS cells, whereas in WT-KRAS cells, MT-KRAS knock-in showed Hh/NF-κB interplay. Collectively, these data suggest that MT-KRAS expression is the key to Hh/NF-κB interplay in PDAC.

The MT-KRAS gene leads to constitutively active KRAS protein (33). Therefore, we decided to examine the expression of KRAS downstream genes and Ras enzymatic activity in PDAC cells treated with IL-1β, TNF-α, Shh alone or in combination with si-KRAS. As a result, KRAS activity was unaltered in WT-KRAS cells but not in MT-KRAS cells. In addition, we did not observe any change in protein expression of nuclear Gli1, nuclear NF-κB, and Shh when MT-KRAS plasmid was knocked into WT-KRAS cells. This might be due to the low level of MT-KRAS protein, which was not sufficient to enhance KRAS activity.

Interestingly, once KRAS was knocked down in Panc-1 cells, TNF-α reduced rather than promoted cell growth. This may be related to the effects of TNF-α, since TNF-α not only activates NF-κB to enhance cell proliferation, but also increases apoptosis. In this study, when KRAS was downregulated, TNF-α was more likely to increase apoptosis than promote cell growth.

Another interesting finding was that when Hh pathway was activated by rh-Shh, other than enhanced nuclear Gli1 and NF-κB expression, Shh itself was also upregulated. Shh is not a target gene of the Hh pathway, so increased Shh expression might be induced by NF-κB expression or some other non-classical Hh activation pathway. Thus, this finding may also be attributed to KRAS mutation in PDAC cells.

In our research, the correlation coefficients between Gli1 and NF-κB, Shh and NF-κB were only slightly over 0.5 in PDAC tissues, and this may be due to inter- and intra-tumor heterogeneity. Hh and NF-κB pathways are crucial in MT-KRAS PDACs, but there are also other pathways involved in PDAC progression. These findings need to be confirmed in a larger patient group.

In a nude mouse xenograft tumor model, tumor developed from SW1990 and Panc-1 cells stimulated with IL-1β, TNF-α, and Shh was significantly promoted compared to control animals, whereas no stimulating effect was observed on tumor development from BxPC-3 cells. This finding suggests that Hh and NF-κB pathways interact in MT-KRAS PDAC. In addition, tumors developed from BxPC-3 were smaller than those from MT-KRAS PDACs, which may suggest the importance of the KRAS gene in PDAC development. Similar alteration on SW1990 cells derived tumors’ Gli1 and NF-kB nuclear protein expression in the nucleus were observed with IL-1β, TNF-α and Shh by IHC assay. However, BxPC-3 derived tumors’ Gli1 and NF-kB protein were also expressed in the nucleus but no alteration was founded on the condition of IL-1β, TNF-α and Shh treatment. This suggests that the progress of BxPC-3 is much more complex than previous thought.

This study may have limitations. Firstly, we found that mut-KRAS knock-in led to significant increases in the Gli1, NF-kappaB, and Shh expression at the RNA level but not at protein level. With the existing data, the mechanisms underlying the differential effects on the RNA and protein levels remain unclear. Secondly, BxPC-3 cells displayed a constitutive intra-nuclear localization of NF-κB/p65, future study on the expression levels of phospho-p65 is needed to help in assessing the effect of mut-KRAS knock-in in WT-KRAS BxPC-3 cells. Thirdly, it was of note in this study that TNF-α treatment in combination with si-KRAS showed a significant increase of apoptosis compared with si-KRAS alone in Panc-1 cells, but not in SW1990 and BxPC-3 cells. At this point, we could not explain why the differential effects exist in various cells. It has also to be pointed out that the significant difference in the total number of cases with or without KRAS mutation may represent a bias in statistical interpretation. This is mainly because that KRAS mutation occurred in a majority of PDAC patients, accounting for as high as 80% (32/40) in this study. Further study with a larger sample size is underway in our center.

In conclusion, KRAS mutation is important for PDAC progression but its effects are not sufficient to initiate PDAC. In addition to the pathways analyzed in this study, KRAS mutations may facilitate activation and positive crosstalk between other pathways in PDAC tumorigenesis, which will need to be examined in future studies.

## Data Availability Statement

The datasets presented in this study can be found in online repositories. The names of the repository/repositories and accession number(s) can be found in the article/**Supplementary Material**.

## Ethics Statement

The studies involving human participants were reviewed and approved by Changhai Hospital Ethics Review Committee. The patients/participants provided their written informed consent to participate in this study.

## Author Contributions

JG, KW, and HX designed the experiments. YuW and DW wrote the manuscript. YuW, YD, XK, XZ, and JJ performed experiments and animal work. DW, YF, YaW, and HW assisted in acquisition, analysis and interpretation of data for the work. WY, YaW, HW, and JJ provided administrative support. JG, KW, HX, YD and DW provided financial support. YuW, DW, and YF revised the manuscript. All authors contributed to the article and approved the submitted version.

## Funding

The present study was supported by grants from the National Natural Science Foundation of China (no. 81472279 and 81272663 to JG; no. 81372482 to KW), Suzhou Science and Technology Development Project (SYSD2016164 to HX), Kunshan Science and Technology Development Project (KS1549 to YD). Education Department Service Local Special Scientific Research Projects of Shaanxi Province, China (20JC030), and Education Department Key Special Scientific Research Projects of Shaanxi Province, China (20JS141 to DW).

## Conflict of Interest

The authors declare that the research was conducted in the absence of any commercial or financial relationships that could be construed as a potential conflict of interest.

## Supplementary Material

Supplementary Figure 1 SW1990 and Panc-1 cell lines are positive for KRAS G12D mutation (MT-KRAS) while BxPC-3 cell line is negative for KRAS mutation (WT-KRAS).

Supplementary Figure 2 Detection of Shh, Gli1 and NF-κB p65 mRNA and protein expression in Panc-1, SW1990 and BxPC-3 cells. **(A)** The mRNA expression analysis of Gli1, NF-κB and Shh. Western blot protein expression analysis was repeated three times (1, 2 and 3). **(C)** Gray analysis of relative protein expression for the Gli1, NF–κB and Shh. *p<0.05; **p<0.01.

Supplementary Figure 3 Original protein Western blots image about stimulatory effects on protein expression of Gli1, NF-κB and Shh on PDAC cell lines treated with IL-1β and TNF-α alone or in combination with si-KRAS.

Supplementary Figure 4 Two kinds of si-KRAS were used to knock down KRAS expression in all three cell lines, Negative control (NC) siRNA was used as control. The mRNA **(A)** and protein **(B, C)** expression were significantly decreased by both the si-KRAS1 and si-KRAS2. The si-KRAS1 silencing was stronger than effects obtained with si-KRAS2, and thus si-KRAS1 was selected for further experiments. *p<0.05; **p<0.01.

Supplementary Figure 5 Original protein Western blots image about stimulatory effects on protein expression of Gli1, NF-κB and Shh on PDAC cell lines treated with Shh as Hh activating ligand treatment alone or in combination with si-KRAS transfection.

Supplementary Figure 6 Effect of the treatment of PDAC cells with Shh as the Hh signaling ligand depending upon the presence or absence of KRAS mutation. Shh upregulated the NF-κB p65 nuclear expression in the SW1990 and Panc-1 cell lines, while these effects were significantly decreased when cells were treated with Shh in combination with si-KRAS. However, no change was observed after treatment of BxPC-3 cell line either with Shh alone or in combination with Shh si-KRAS.

Supplementary Figure 7 Plasmid expressing KRAS (G12D) mutation (MT-KRAS plasmid) was constructed and transfected into BxPC-3 cell line. The mRNA **(A)** and protein expression **(B, C)** were tested by qPCR and Western blotting, respectively. As shown, MT-KRAS plasmid transfection significantly increased KRAS expression in BxPC-3. *p<0.05; **p<0.01.

Supplementary Figure 8 The effect of WT-KRAS BxPC-3 PDAC cell line treatment with IL-1β, TNF-α, Shh, in combination with mut-KRAS transfections on Gli1, NF-κB and Shh mRNA expression. All data were obtained in three independent experiments. *p<0.05; **p<0.01.

Supplementary Figure 9 Original protein Western blots image about effect of IL-1β, TNF-α, Shh treatment in combination with si-KRAS on KRAS down-stream molecules (p-/t-ERK1/2 and p-/t-AKT1) and Ras enzymatic activity in the MT-KRAS cell lines (SW1990 and Panc-1) and WT-KRAS cell line (BxPC-3). The protein expression of p-/t-ERK1/2 and p-/t-AKT1 at Shh treatment in combination with si-KRAS treatment **(A)**; The effect of combined IL-1β or TNF-α with si-KRAS transfection on p-/t-ERK1/2 and p-/t-AKT1 **(B)**; Ras enzymatic activity at Shh treatment in combination with si-KRAS treatment **(C)**; The effect of combined IL-1β or TNF-α with si-KRAS transfection on Ras enzymatic activity **(D)** [a. SW1990, b. Panc-1, c. BxPC-3]. All data were obtained in three independent experiments

Supplementary Figure 10 The nuclear protein expression of Gli1 and NF-κB p65 in mice inoculated with MT-KRAS cells compared with those inoculated with WT-KRAS cells. All data were obtained in three independent experiments. *p<0.05; **p<0.01.

## Abbreviations

H&E, hematoxylin and eosin; Hh, Hedgehog; si-KRAS, Human KRAS small interfering RNA; IHC, immunohistochemical; IL-1β, interleukin-1-beta; ip, intraperitoneally; DAB, liquid 3,3’-diaminobenzidine; NF-κB, nuclear factor-kappa B; PDAC, pancreatic ductal adenocarcinoma; PBS, phosphate-buffered saline; PVDF, polyvinylidene difluoride membranes; TNF-α, tumor necrosis factor-alpha.
