# Supplementary figures and images for: Positive Crosstalk Between Hedgehog and NF-κB Pathways Is Dependent on KRAS Mutation in Pancreatic Ductal Adenocarcinoma
Source: Front Oncol. 2021 May 11;11:652283. doi: 10.3389/fonc.2021.652283 (PMC8144522; doi:10.3389/fonc.2021.652283)

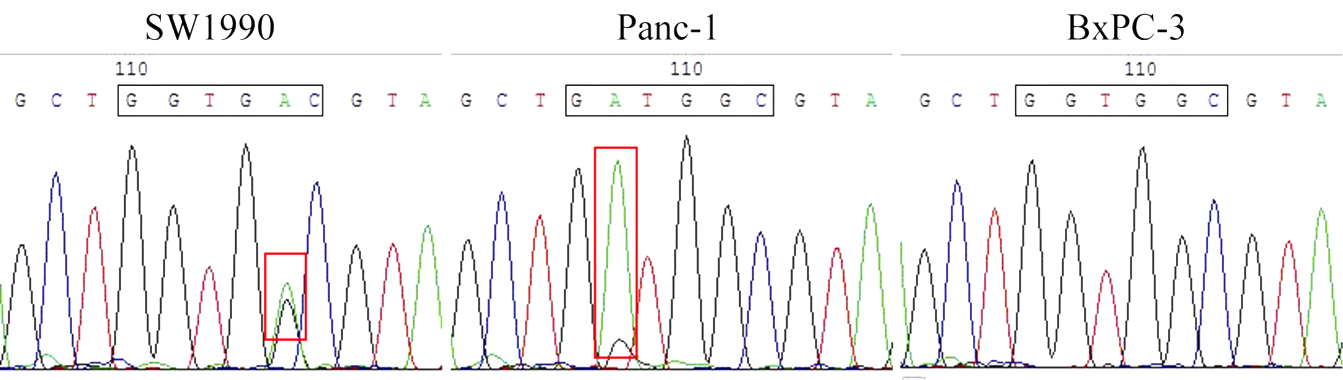

Supplement: Supplementary Figure 1 — SW1990 and Panc-1 cell lines are positive for KRAS G12D mutation (MT-KRAS) while BxPC-3 cell line is negative for KRAS mutation (WT-KRAS). [file Image_1.tif]

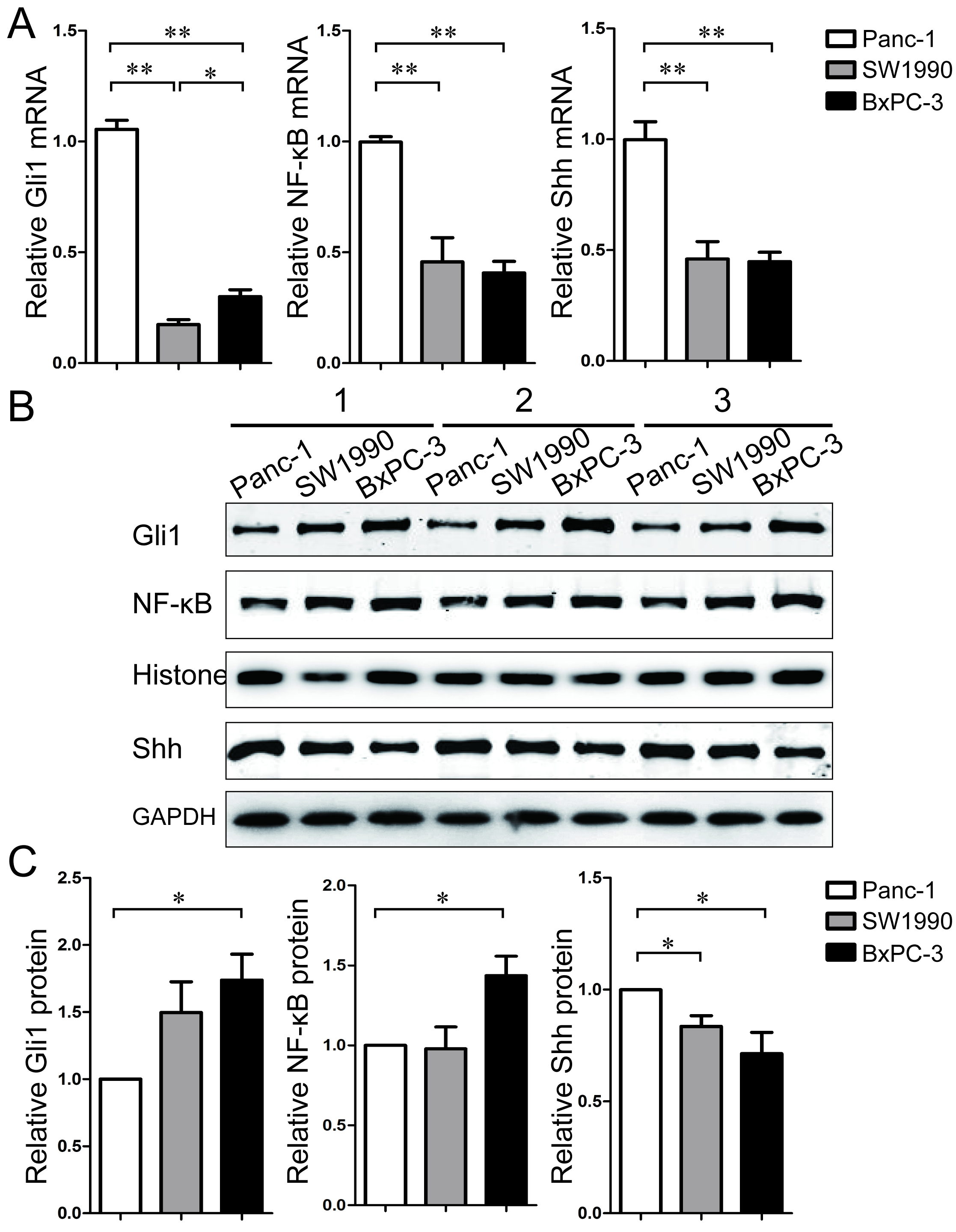

Supplement: Supplementary Figure 2 — Detection of Shh, Gli1 and NF-κB p65 mRNA and protein expression in Panc-1, SW1990 and BxPC-3 cells. (A) The mRNA expression analysis of Gli1, NF-κB and Shh. Western blot protein expression analysis was repeated three times (1, 2 and 3). (C) Gray analysis of relative protein expression for the Gli1, NF–κB and Shh. *p<0.05; **p<0.01. [file Image_2.tif]

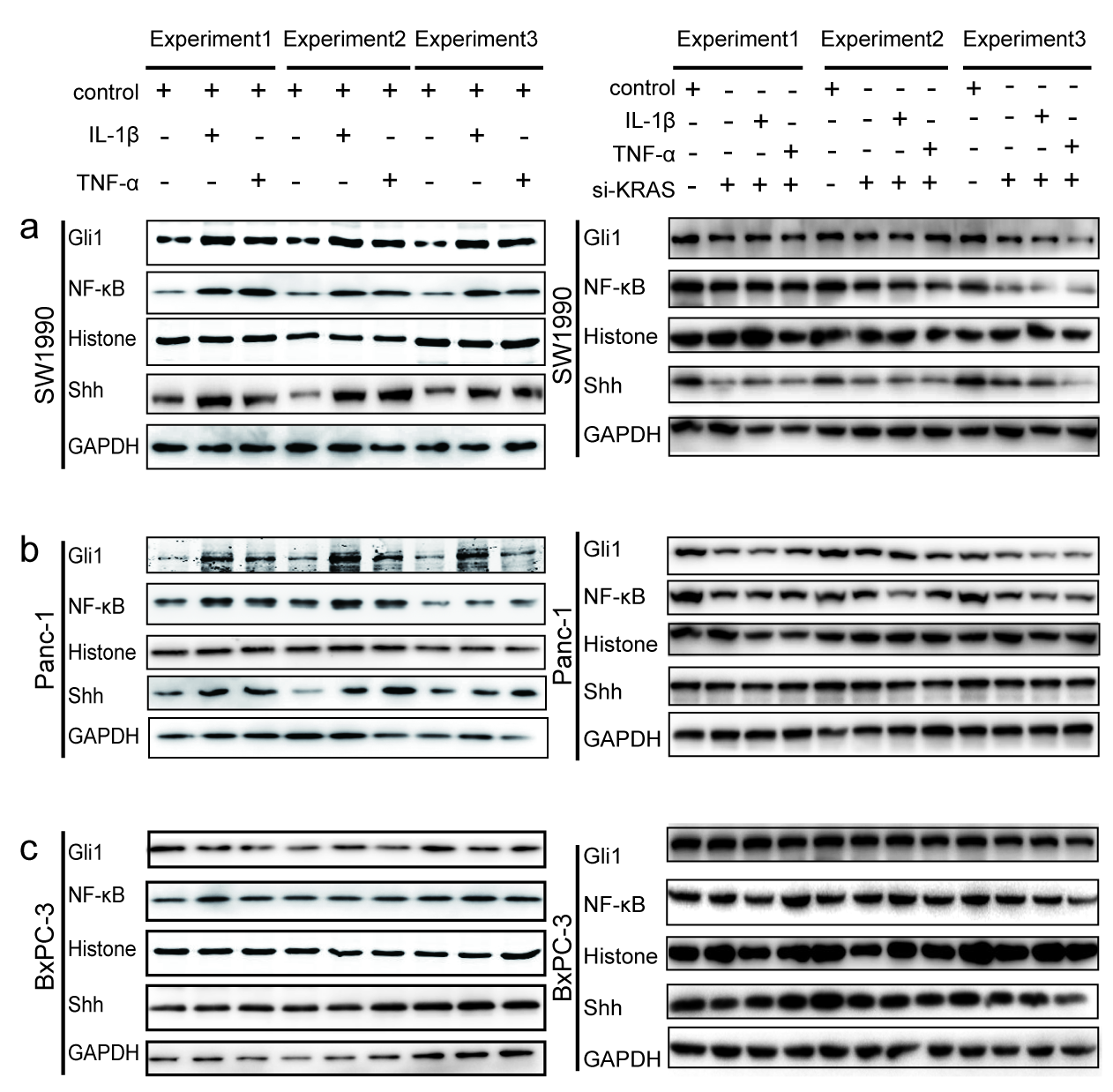

Supplement: Supplementary Figure 3 — Original protein Western blots image about stimulatory effects on protein expression of Gli1, NF-κB and Shh on PDAC cell lines treated with IL-1β and TNF-α alone or in combination with si-KRAS. [file Image_3.tif]

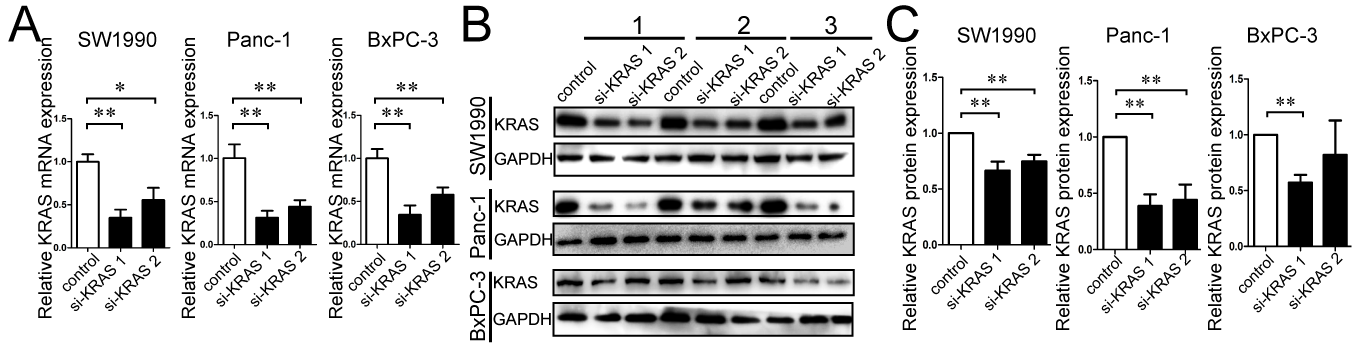

Supplement: Supplementary Figure 4 — Two kinds of si-KRAS were used to knock down KRAS expression in all three cell lines, Negative control (NC) siRNA was used as control. The mRNA (A) and protein (B, C) expression were significantly decreased by both the si-KRAS1 and si-KRAS2. The si-KRAS1 silencing was stronger than effects obtained with si-KRAS2, and thus si-KRAS1 was selected for further experiments. *p<0.05; **p<0.01. [file Image_4.tif]

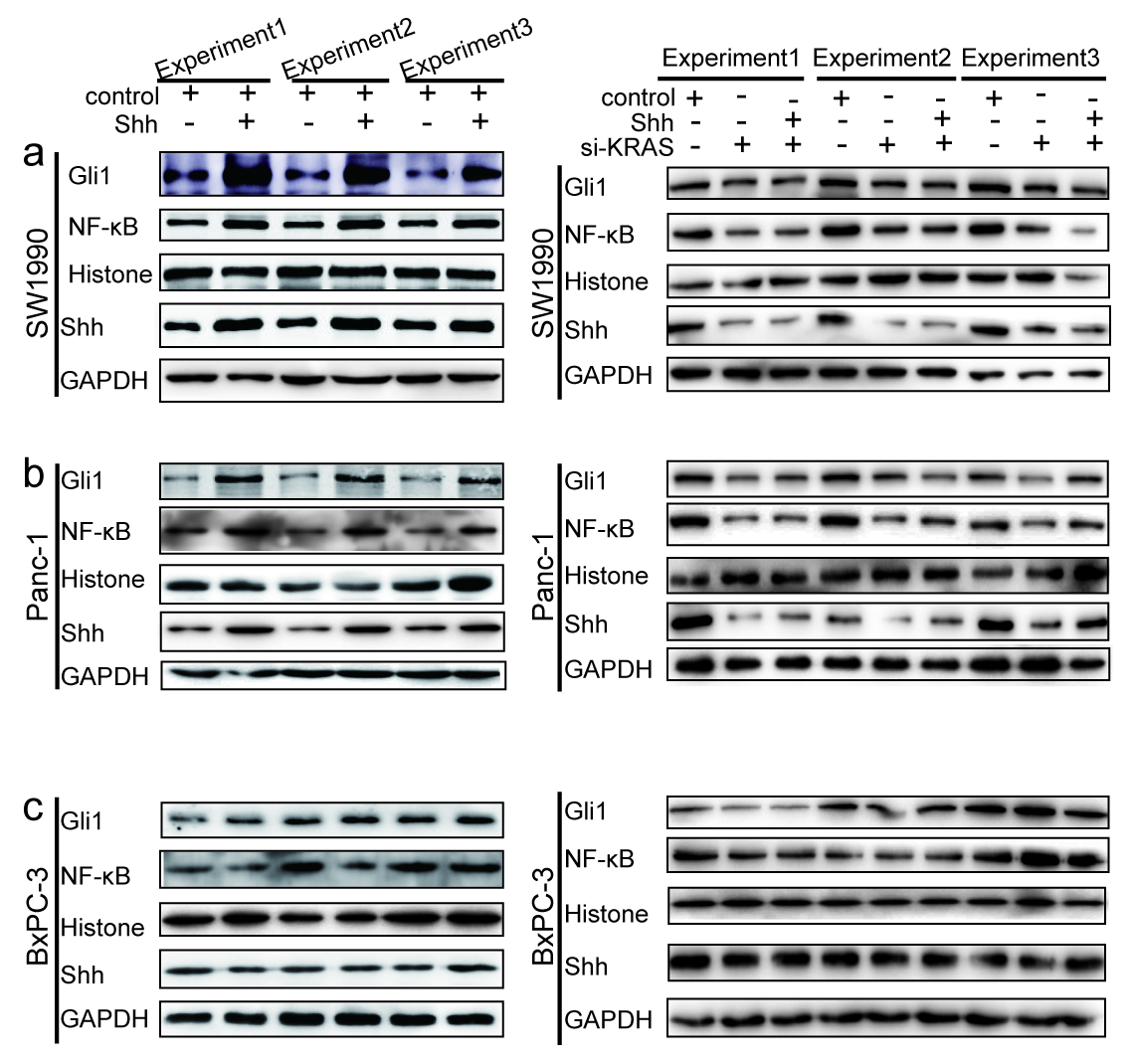

Supplement: Supplementary Figure 5 — Original protein Western blots image about stimulatory effects on protein expression of Gli1, NF-κB and Shh on PDAC cell lines treated with Shh as Hh activating ligand treatment alone or in combination with si-KRAS transfection. [file Image_5.tif]

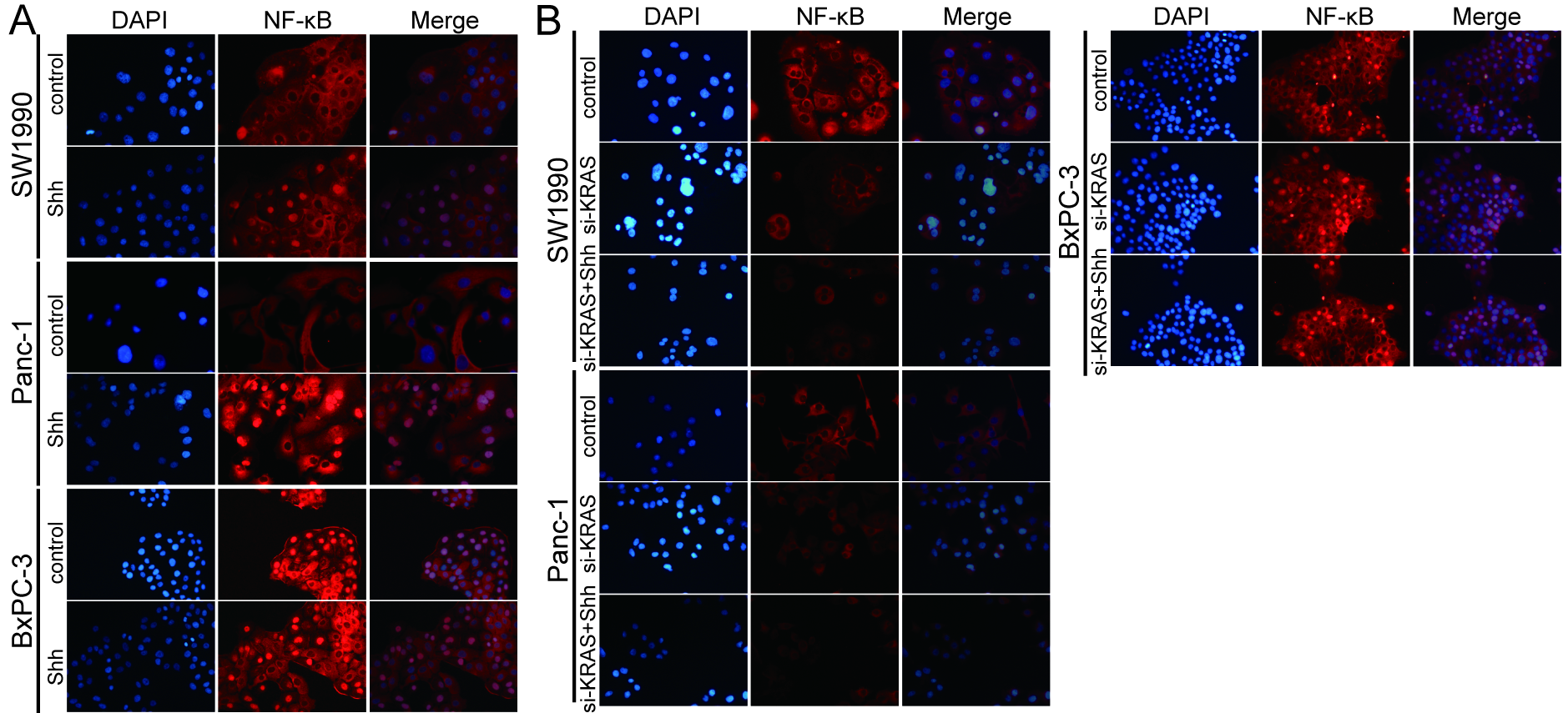

Supplement: Supplementary Figure 6 — Effect of the treatment of PDAC cells with Shh as the Hh signaling ligand depending upon the presence or absence of KRAS mutation. Shh upregulated the NF-κB p65 nuclear expression in the SW1990 and Panc-1 cell lines, while these effects were significantly decreased when cells were treated with Shh in combination with si-KRAS. However, no change was observed after treatment of BxPC-3 cell line either with Shh alone or in combination with Shh si-KRAS. [file Image_6.tif]

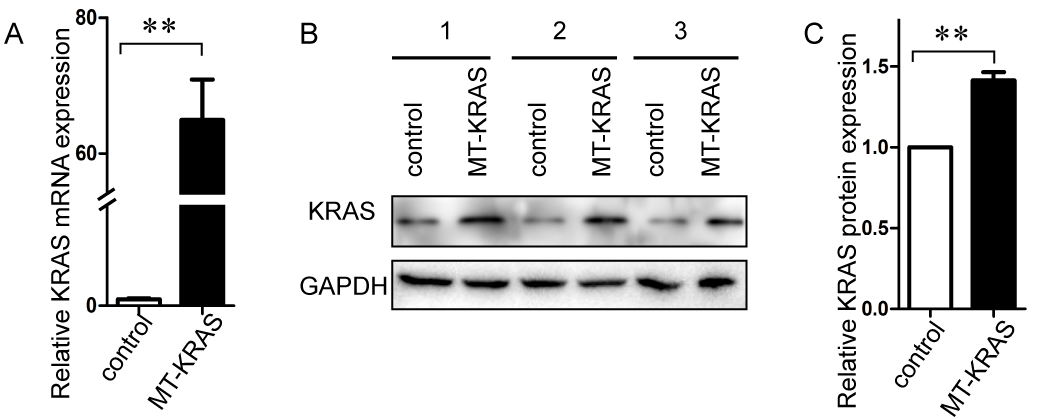

Supplement: Supplementary Figure 7 — Plasmid expressing KRAS (G12D) mutation (MT-KRAS plasmid) was constructed and transfected into BxPC-3 cell line. The mRNA (A) and protein expression (B, C) were tested by qPCR and Western blotting, respectively. As shown, MT-KRAS plasmid transfection significantly increased KRAS expression in BxPC-3. *p<0.05; **p<0.01. [file Image_7.tif]

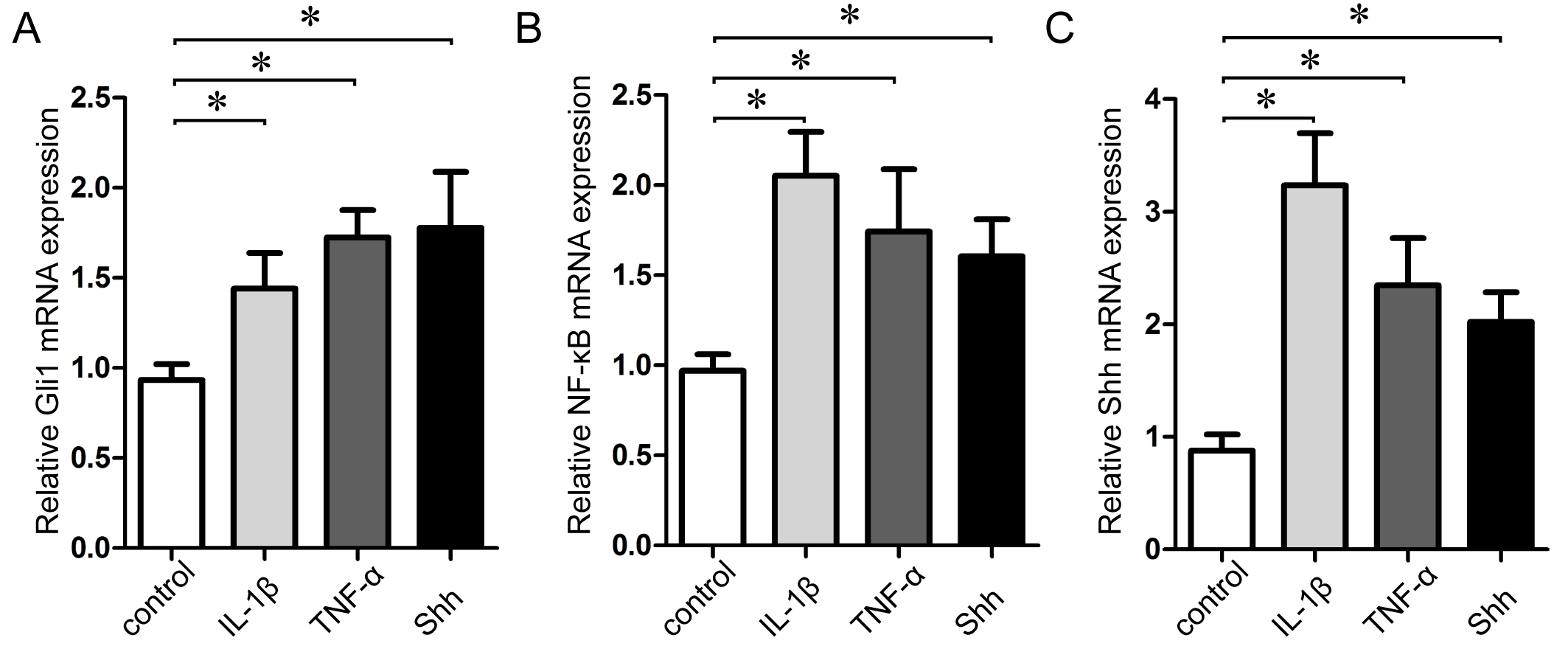

Supplement: Supplementary Figure 8 — The effect of WT-KRAS BxPC-3 PDAC cell line treatment with IL-1β, TNF-α, Shh, in combination with mut-KRAS transfections on Gli1, NF-κB and Shh mRNA expression. All data were obtained in three independent experiments. *p<0.05; **p<0.01. [file Image_8.tif]

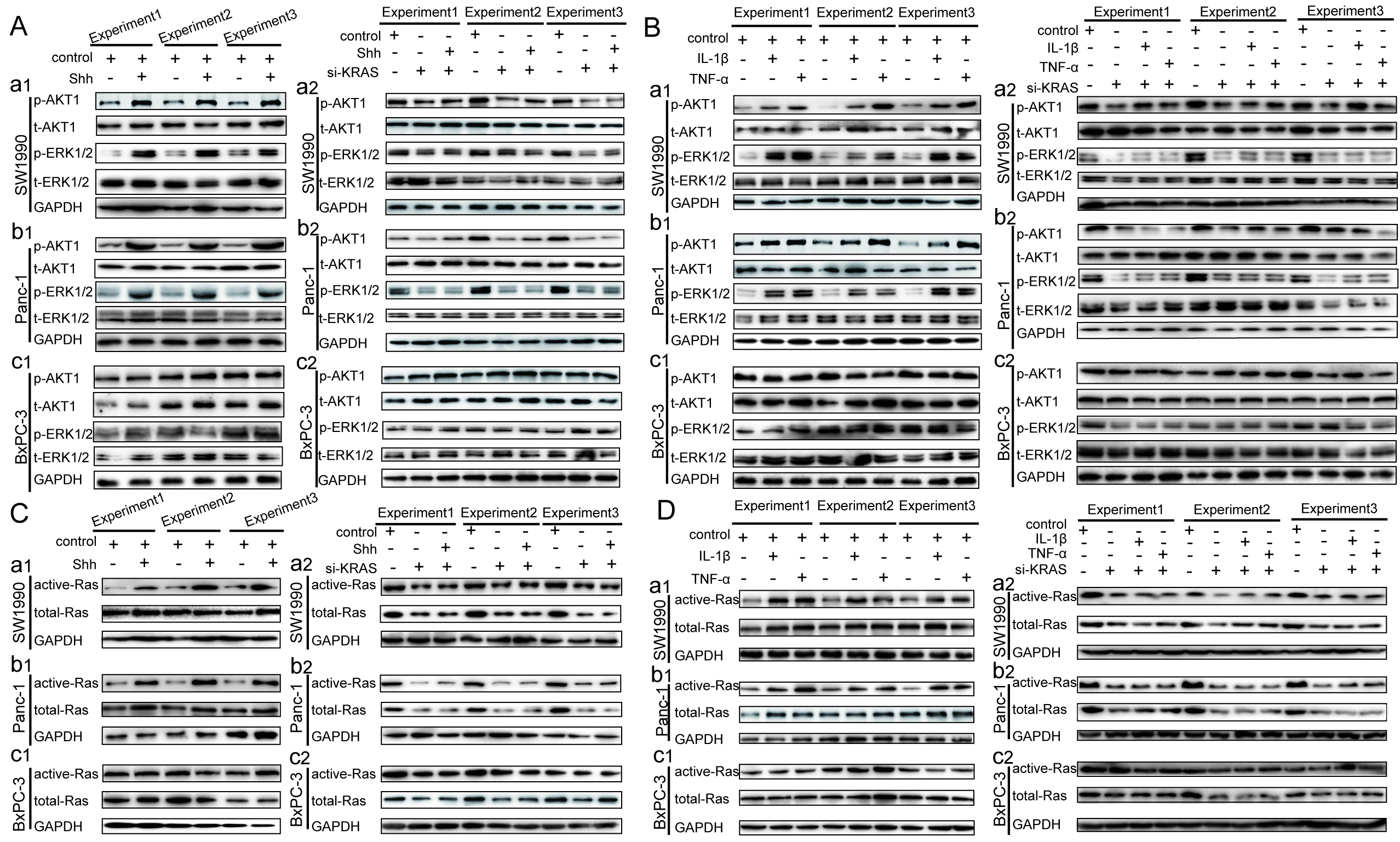

Supplement: Supplementary Figure 9 — Original protein Western blots image about effect of IL-1β, TNF-α, Shh treatment in combination with si-KRAS on KRAS down-stream molecules (p-/t-ERK1/2 and p-/t-AKT1) and Ras enzymatic activity in the MT-KRAS cell lines (SW1990 and Panc-1) and WT-KRAS cell line (BxPC-3). The protein expression of p-/t-ERK1/2 and p-/t-AKT1 at Shh treatment in combination with si-KRAS treatment (A); The effect of combined IL-1β or TNF-α with si-KRAS transfection on p-/t-ERK1/2 and p-/t-AKT1 (B); Ras enzymatic activity at Shh treatment in combination with si-KRAS treatment (C); The effect of combined IL-1β or TNF-α with si-KRAS transfection on Ras enzymatic activity (D) [a. SW1990, b. Panc-1, c. BxPC-3]. All data were obtained in three independent experiments [file Image_9.tif]

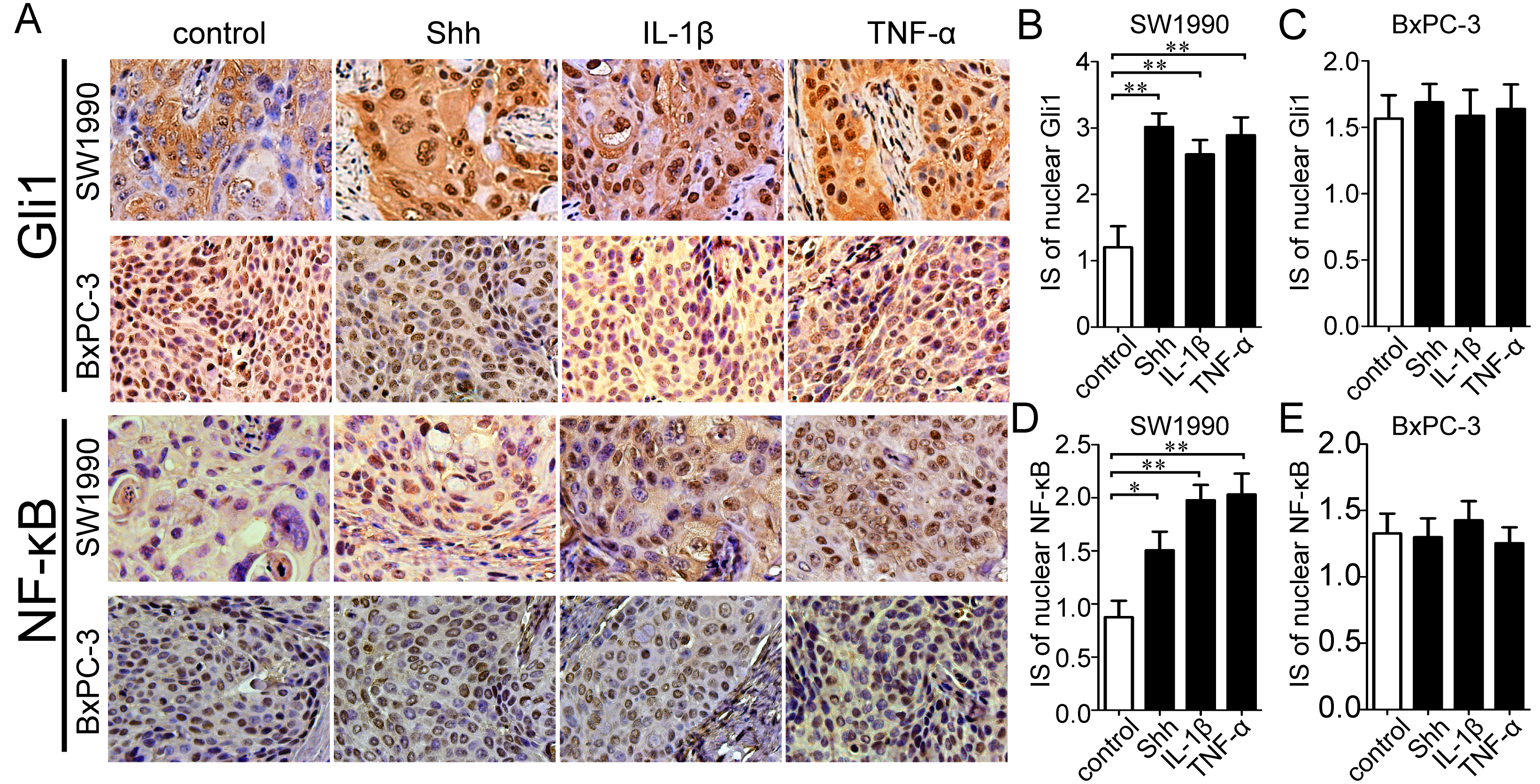

Supplement: Supplementary Figure 10 — The nuclear protein expression of Gli1 and NF-κB p65 in mice inoculated with MT-KRAS cells compared with those inoculated with WT-KRAS cells. All data were obtained in three independent experiments. *p<0.05; **p<0.01. [file Image_10.tif]
